# Perioperative Challenges and Surgical Treatment of Large Simple, and Infectious Liver Cyst - A 12-Year Experience

**DOI:** 10.1371/journal.pone.0076537

**Published:** 2013-10-02

**Authors:** Yuichiro Maruyama, Koji Okuda, Toshiro Ogata, Masafumi Yasunaga, Hiroto Ishikawa, Yusuke Hirakawa, Kenjiro Fukuyo, Hiroyuki Horiuchi, Osamu Nakashima, Hisafumi Kinoshita

**Affiliations:** 1 Department of Surgery, University School of Medicine, Kurume, Japan; 2 Department of Pathology, University School of Medicine, Kurume, Japan; UNIFESP Federal University of São Paulo, Brazil

## Abstract

**Background:**

Cystic lesions of the liver consist of a heterogeneous group of disorders that can present diagnostic and therapeutic challenges.

**Methods:**

A retrospective review of all medical records of adult patients diagnosed with large (>7 cm) cystic lesions of the liver between January 2000 and December 2011, at Kurume University Hospital. Cases with polycystic disease were excluded.

**Results:**

Twenty three patients were identified. The mean size was 13.9 cm (range, 7-22cm). The majority of simple cysts were found in women (females: males, 2: 21). In 19 patients, the cyst was removed surgically by wide deroofing (laparoscopically in 16 cases, combined with ethanol sclerotherapy in 13 cases). Infection of the liver cyst occurred in one patient, who later underwent central bi-segmentectomy.

**Conclusion:**

Simple large cysts of the liver can be successfully treated by laparoscopic deroofing and alcohol sclerotherapy. Large hepatic cyst considered to need drainage should be removed surgically to avoid possible infection.

## Introduction

Hepatic cysts are classically divided into parasitic and non-parasitic types, with the latter being the most prevalent worldwide. Large hepatic cysts tend to be symptomatic, and may cause complications more often than smaller ones [1].

Liver cysts are also classified as true or false, based on the presence or absence of epithelial lining. True cysts include congenital cysts, simple cysts, cysts caused by *Echinococcusgranulosis* and multilocularis tapeworms, neoplastic cysts (e.g., cystadenoma, cystadenocarcinoma, cyst sarcoma, squamous cell carcinoma, and metastatic ovarian, pancreatic, colon, renal and neuroendocrine cancers), and biliary duct-related cysts (Caroli’s disease, bile duct duplication, and peribiliary cysts). False cysts may be caused by spontaneous intrahepatic hemorrhage, post-traumatic hematoma, or intrahepatic biloma.

The pathogenesis of liver cysts is not clear. Simple liver cysts are congenital. They are generally stable in size overtime, but may slowly increase in size and occasionally become symptomatic due to mass effect, rupture, hemorrhage, or infection [2]. One European study found 1,235 liver cysts in more than 26,000 patients undergoing upper abdominal ultrasonography, with a calculated incidence of 4.75%. Hepatic cysts are encountered more often in female than male patients, with an estimated female to male ratio of 1.5:1. Furthermore, liver cysts are symptomatic more often in women than in men [3].

Treatment of simple liver cysts include laparoscopic deroofing and alcohol sclerotherapy [4]. Wide deroofing and cyst resection are associated with low incidence of cyst recurrence or complications [5].

We report herein the clinical features of large cystic liver disease, together with the selected therapeutic approaches and outcome.

## Methods

We performed a retrospective review of all medical records of adult patients diagnosed with large (>7cm) cystic lesions of the liver between January 2000 and December 2011, at Kurume University Hospital, Kurume, Japan.

Data were extracted from the hospital records for demographics, clinical presentation, size and location of the lesions. When histology was available, the following histopathological criteria were used for definitive diagnosis of simple cyst: 1) an outer layer of a thin dense fibrous tissue, and 2) an inner-epithelial lining consisting of a single layer of cuboidal or columnar epithelium. The study protocol was approved by the Human Ethics Review Committee of Kurume University and informed consent was given by the patients to store the clinical information in the hospital.

The correlation of different treatment types with morbidity were analyzed by fisher’s exact test. Differences were considered significant at P <0.05. 

## Results

Over the 11-year study period, 23 adult patients with large cystic lesions of the liver were diagnosed at our hospital. Seventeen patients (74%) with simple cysts were referred because of abdominal symptoms, which consisted of right upper quadrant or epigastric pain or discomfort and feeling of abdominal distention. Table 1 summarizes the demographic and clinical data of these patients. The American Society of Anethesiologists physical status classification system classification was class 1 in all patients.

**Table 1:** Demographic and clinical data of 23 patients with large liver cysts diagnosed at our hospital between 1999 and 2011.

Males/females	3/20
Mean age (years) (range)	63.4 (27-78)
Symptomatic/asymptomatic (Pain: nausea: abdominal distension)	17/6 (14:1:2)
Mean size (cm) (range)	13.9 (7-22)
Single/multiple (%)	12/11 (52%/48%)
Location (right hemiliver/left hemiliver/both)	11/4/8
Treatment (deroofing/liver resection)	19/4
Alcohol sclerosis	13
Postoperative malignancy	2

### Simple cyst

**Figure 1:**
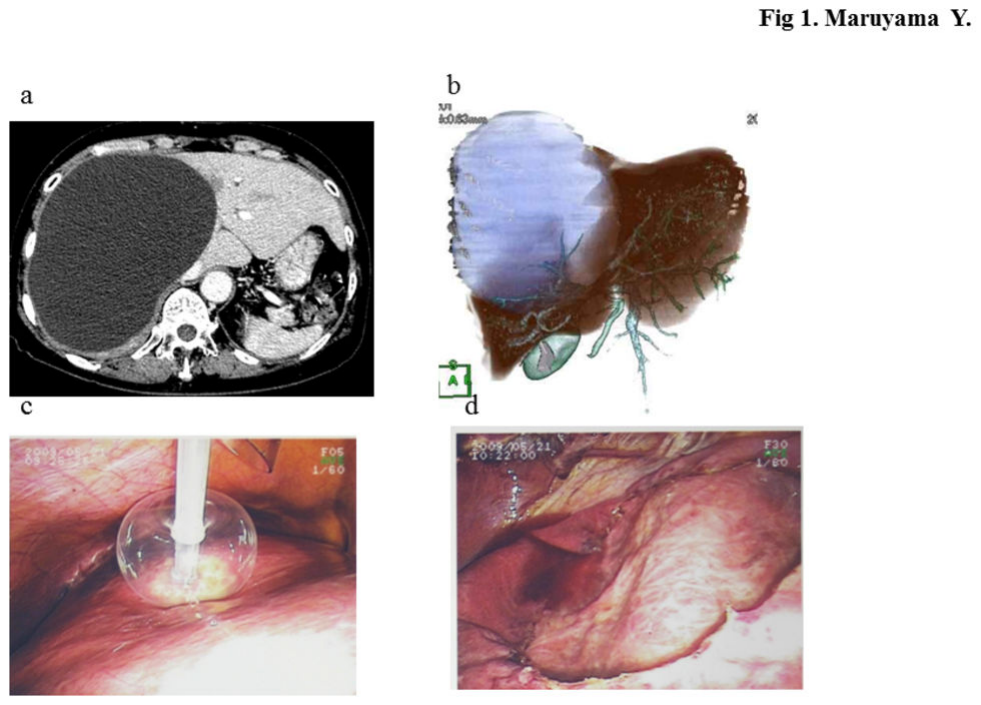
Simple hepatic cyst in a 64-year-old woman. a) Abdominal CT scan shows a well-defined water attenuation lesion in the right hepatic lobe. (b) 3D-image showed a cyst capacity of 1800 ml. Note the Glisson’s pedicle in the base. (c) Laparoscopic aspiration and ethanol sclerosis using sand balloon catheter. (d) Wide unroofing in the right hepatic lobe.

Nineteen of the 23 (82.6%) patients were eventually managed surgically by deroofing (with or without ethanol sclerosis) (Table 2). The standard surgery used in our department is laparoscopic deroofing. When the abdominal computed tomography (CT) scan showed a well-defined water attenuation lesion (Figure 1a), we used the 3D image created by the workstation to measure the cyst capacity (Figure 1b). Surgery included cyst wall puncture by sandballoon catheter (Figure 1c), aspiration, and injection of anhydrous alcohol using a volume equivalent to 10% of the cyst capacity. Ten minutes after the injection, ethanol was removed, followed by deroofing using energy device (Figure 1d).

**Table 2:** Comparison of clinical findings.

	Deroofing and liver resection				
	Number (%)	Hospital stay (days) (range)	Operative time (mean) (range)	Morbidity (%) complication	Recurrence (%)
Operative Procedures					
**Deroofing**	19 (82.6)			2 (12.5)	3 (18.7)
Laparoscopic deroofing	16 (69.6)	17.8 (10–38)	165.8 (90-270)	2 (12.5) bile leakage	3 (18.7)
Alcohol sclerosis (yes)	13				2 (15.4)
Alcohol sclerosis (no)	3				1 (33.3)
Open deroofing	3	23 (20-26)		0	0
**Hepatic resection**	4 (17.4)	41.8 (30–58)	400 (390-410)	2 (50) atelectasis	0

Four of the 23 (17.4%) patients also required segmentectomy or lobectomy of the liver (Table 2). The reason for the latter two procedures was suspected cystadenoma by radiography. The liver resection procedures included posterior segmentectomy, right lobectomy, left lobectomy, and central bi-segmentectomy.

### Infectious liver cyst

**Figure 2:**
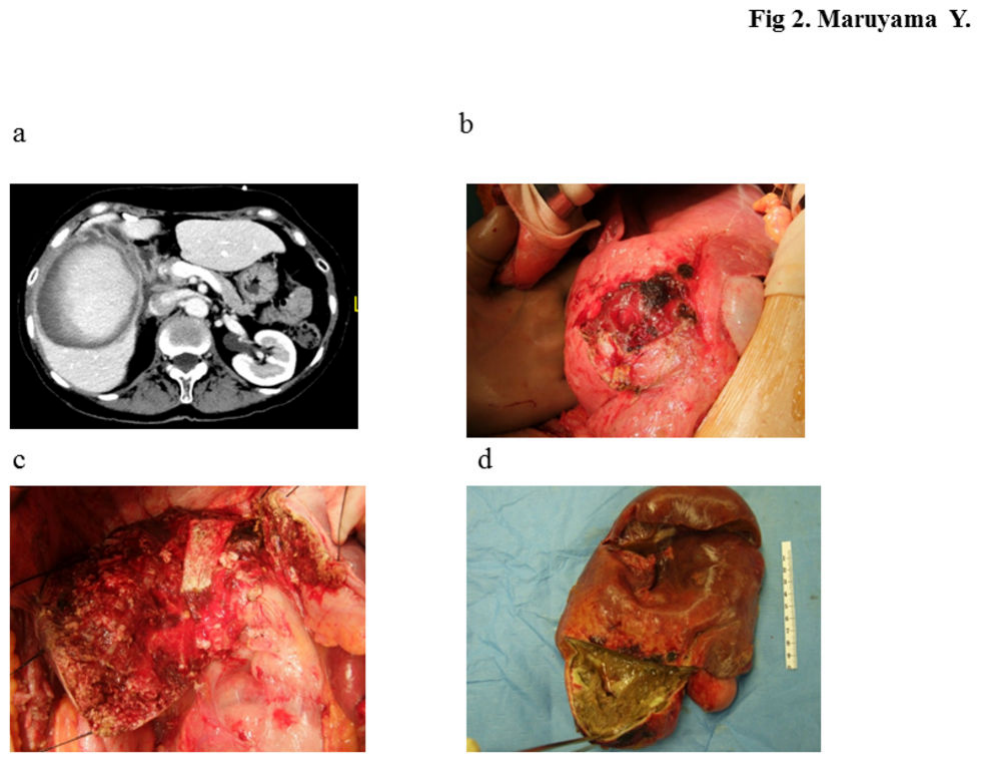
Infectious liver cyst in a 71-year-old woman. (a) Abdominal CT scan showed enhanced cystic wall. (b) Laparotomy view. (c) Intraoperative photograph of post-central hepatectomy. (d) Surgical sample: note the presence of an abscess separated by a septum from the tumor area.

In one of our patients, infectious liver cyst developed 2 weeks after cyst drainage. The patient underwent drainage of a huge liver cyst, but this was followed by fever and liver abscess was diagnosed, which was probably caused by infection with the drainage tube (Figure 2a). Radiographic examination showed suspicious cystadenoma, and was scheduled for removal by hepatectomy. However, abdominal computed tomography (CT) scan showed part of the drainage tube in the cystic lesion, thus the patient was treated first with intravenous antibiotics, and later underwent central bi-segmentectomy (Figure 2b-c). Histopathological examination showed a simple hepatic cyst (Figure 2d).

The mean operation time was 165.8 min (range, 90 to 270) for laparoscopic deroofing (Table 2). No operative death was encountered in the present series. The morbidity rate was significant after laparoscopic deroofing (12.5%) and hepatic resection (50%). The mean hospital stay was 17.8 days (range, 10-38 days) for patients who underwent laparoscopic deroofing and 41.8 days (range, 30 to 58 days) for those who underwent liver resection (Table 2). All patients were followed-up for a minimum of 5 years (range, 5-10 years). Recurrence of the cysts was noted in 3 of 19 patients who underwent follow-up imaging after deroofing. None of the patients who developed recurrence required liver resection. There were no significant difference in the correlation of different treatment types with morbidity. Two patients died of hepatobiliary malignancy; one developed hilar bile duct cancer five years after discharge, and the other developed pancreatic cancer 10 years after surgery (Table 1).

## Discussion

Simple hepatic cysts are benign developmental lesions that do not communicate with the biliary tree. Hepatic cysts are common and presumed to be present in 2.5% of the population. They are more often discovered in women and are almost always asymptomatic. Histopathologically, true hepatic cysts contain serous fluid and are lined by nearly imperceptible wall consisting of cuboidal epithelium, identical to that of bile ducts, and a thin underlying rim of fibrous stroma. On non-enhanced computed tomography (CT) scans, hepatic cysts appear as homogeneous and hypoattenuated lesions with no enhancement of the wall or content after intravenous administration of contrast material [6]. Although simple hepatic cysts are found in about 1% of necropsied adults, very few are large, and even fewer cause symptoms.

In the majority of our patients, the diagnosis of simple hepatic cyst was established by abdominal ultrasonography, which demonstrated characteristic features of non-complicated simple cysts.

Several therapeutic approaches have been described for simple hepatic cysts, including simple aspiration, with or without injection of sclerosing agent, internal drainage with cyst jejunostomy, wide deroofing, and variable extent of hepatic resection. As described in previous reports, simple aspiration in our series was associated with high failure rate, whereas low incidence of cyst recurrence and complications were noted after wide deroofing or cyst resection. The reported recurrence rate after laparoscopic deroofing of solitary simple cysts ranges from 0% to 14.3%, while the morbidity rate ranges from 0% to 25% [7–10]. The laparoscopic approach has proved safe, achieving wide deroofing without the use of debilitating incision. Thus, surgical therapy seems to be the only definitive treatment for symptomatic simple cysts, and that laparoscopic deroofing may be the procedure of choice for accessible cysts.

Both laparoscopic deroofing and cyst sclerosis are effective in partial or complete obliteration of the cyst and in the relief of symptoms associated with hepatic cysts. It is important, however, to rule out cystadenoma, malignancy, biliary communication and infection. Aspiration followed by sclerotherapy with alcohol is also used. Furthermore, laparoscopic deroofing of hepatic cysts has been attempted with satisfactory results. Laparoscopic deroofing is usually carried out by a 4-port technique with the patient in the supine position. Based on the above background, alcohol sclerosis seems effective for laparoscopic deroofing, with the advantage of low incidence of complications. One major advantage of surgery compared with cyst sclerosis is direct visualization of the cyst interior.

The mean hospital stay in our study was relatively long compared with that reported in a previous study [11]. The difference likely represents differences in patient management under two different health systems; our patients were examined and underwent various tests and imaging studies after admission to the hospital. However, the clinical results, e.g., morbidity and mortality rates, were similar to previous report [12]. Liver resection was associated with a higher morbidity rate, but no mortality. Based on these results, we believe that liver resection for cystic lesions is safe and efficacion [13].

The initial treatment of infectious liver cyst is drainage, as it helps exclude cystadenocarcinoma, and thus allows the next procedure of hepatectomy. To our knowledge, there are no reports of resection of liver abscess after drainage of huge cyst.

Two of our patients died of hepatobiliary malignancy. The first developed hilar bile duct cancer five years after treatment of the simple hepatic cyst, while the second developed pancreatic cancer 10 years later.

Intraductal papillary mucinous neoplasm (IPMN) is reported to coexist with synchronous malignant diseasein approximately 27% of the cases [14]. To our knowledge, there are no reports of malignancy after deroofing of large hepatic cysts, though such information is important to be documented.

In conclusion, large symptomatic hepatic cysts are often simple cysts, though infrequently are hepatobiliary cystadenomas. Laparoscopic deroofing is the procedure of choice for large symptomatic simple cysts. Large hepatic cysts considered to need drainage should be rather removed surgically to avoid possible infection.
